# Supplementary material for: Traffic accident risk prediction based on deep learning and spatiotemporal features of vehicle trajectories
Source: PLoS One. 2025 May 2;20(5):e0320656. doi: 10.1371/journal.pone.0320656 (PMC12048164; doi:10.1371/journal.pone.0320656)
Supplement: Attachment — Submitted filename: Response to Reviewers_.docx [file pone.0320656.s002.docx]

**Reviewer 1:**

Thank you very much for your valuable comments on this article during your busy schedule. I have made careful modifications according to your requirements, and I will reply to your questions below.

**1.Question:**All formulas in this article lack numbering to ensure that the main text and formulas comply with the journal's required format.

**Answer:**All formulas have been modified according to the journal's requirements.

**2.Question:**Lack of comparative experiments with state-of-the-art methods.
**Answer:**The state-of-the-art methods have been studied, and comparative experiments have been conducted.

**3.Question:**Lack of visualization results for trajectory prediction and risk prediction.
**Answer:**Trajectory prediction and risk prediction visualizations have been added.
**4.Question:**The innovative part of this article is not clear enough. Please present it in sections after the introduction.
**Answer:**Introductions have been made in the introduction and subsequent chapters.
**5.Question:**Please provide the parameters of the model designed in this article and the hyperparameters used during training.
**Answer:**The model parameters and hyperparameters have been provided during the experimental process.

**6.Question:**Insufficient summarization of existing research in the Introduction, it is recommended to cite " Research on multi-lane energy-saving driving strategy of connected electric vehicle based on vehicle speed prediction" and " Continual driver behavior learning for connected vehicles and intelligent transportation systems: Framework, survey and challenges"

**Answer:**Your valuable suggestions have been adopted.

**Reviewer 2:**

Thank you very much for your valuable comments on this article during your busy schedule. I have made careful modifications according to your requirements, and I will reply to your questions below.

**1.****Question:** The abstract is not concise enough to sketch the entire theme, in particular, the results of the manuscript.

**Answer****:**The redundant parts of the manuscript have been simplified.

2.**Question:**Furthermore, the introduction section needs considerable effort (concise and brief). The problem being investigated should be described clearly, but before that, the field of research should be made clearer. Furthermore, briefly describe the major contributions in bullet form, just before the organization paragraph.
**Answer:**The revisions have been made as requested.
**3.Question:**I suggest summarizing the related works into a table with respect to their characteristics. The authors should put their proposal into this table for easy comparison. Furthermore, I have listed some related works that authors should consider for evaluation of their work.
**Answer:**Thank you for your valuable suggestions. I have implemented them.
**4.Question:** Do the results shown in various figures refer to a single run or multiple runs (average)? In the latter case, I will suggest adding standard deviation bars. The reason behind this is to ensure that the results overlap with the closest rivals or not.
**Answer:**The results from multiple runs have been provided. I have implemented your suggestions and added standard deviation bars to some of the images.
**5.Question:**Furthermore, the evaluation metrics should be briefly described in the experimental section. Moreover, add further details on how the experiments were conducted. Which tool was used to implement the algorithms? Similarly, system and resource characteristics should be added for clarity.
**Answer:**The relevant details have been explained during the experiment.
**6.Question:**Some sections have repeated text or the discussion is too detailed and should be reduced. In fact, the paper is quite wordy, and the authors should make significant efforts to make it concise and short.
**Answer:**The redundant content has been simplified.
**7.Question:**Add further details on how simulations were conducted. Perhaps add a flowchart that clearly identifies how the entire system works.

**Answer:**The model operation flowchart has been added.

8.**Question:**The conclusion section also needs significant revisions. It should briefly describe the findings of the study and some more directions for further research.
**Answer:**The conclusion has been refined.
**9.Question:**Proofread the article to ensure appropriate use of English grammar, tenses, and punctuation. Longer sentences should be broken out into smaller ones. There are also some linguistic issues that should be corrected. The use of article "the" is redundant and somewhere missing.
**Answer:**The entire text has been polished.
**10.Question:**I suggest adding a brief description of each figure in its caption.

**Answer:**A brief description has been provided for each image.
**11.Question:**Authors should describe the scalability of the proposed algorithms. Is the system scalable? If yes, under what assumptions and conditions?
**Answer:**Scalability-related experiments have been conducted.
**12.Question:**Add further details on how simulations were conducted. Perhaps add a flowchart that clearly identifies how the entire system works. The evaluation metrics should be briefly described in the experimental section. Moreover, add further details on how simulations were conducted. Similarly, system and resource characteristics could be added to tables for clarity.
**Answer:**The model operation flowchart has been added.
**13.Question:**More experiments should be performed to study the scalability of the proposed algorithms.
**Answer:**Scalability-related experiments have been conducted.
**14.Question:** How can the proposed system be implemented in practice? I believe authors should consider this instead of giving unnecessary details of various systems and concepts.

**Answer:**The methods for how the model is applied in practice have been added.
**15.Question:**The paper makes no mention of how the proposed algorithms and system will be implemented in practice, what the algorithm's computational cost is, or how it can be integrated into a real-world system.
**Answer:**An overview has been provided in the paper.
**16.Question:**Furthermore, what is being advanced in the state of the art and what originality is being introduced to the traffic prediction literature are both lacking in novelty. There is relatively little scientific contribution because this does not offer a new theory or model that is not already available.
**Answer:** The latest models have been studied, and comparative experiments have been conducted.

**17.Question:** Furthermore, each and every mathematical notation should be explained. I suggest adding a table that describes all the mathematical notations with brief definitions.

**Answer:** All mathematical formulas have been declared.
