# Supplementary material for: Traffic accident risk prediction based on deep learning and spatiotemporal features of vehicle trajectories
Source: PLoS One. 2025 May 2;20(5):e0320656. doi: 10.1371/journal.pone.0320656 (PMC12048164; doi:10.1371/journal.pone.0320656)
Supplement: Attachment — Submitted filename: Response_to_Reviewers__auresp_2.docx [file pone.0320656.s003.docx]

**Reviewer 2:**

Thank you very much for your valuable comments on this article during your busy schedule. I have made careful modifications according to your requirements, and I will reply to your questions below.

**Answer:**This study has made modifications to the repeated citations and has followed your suggestions to categorize the relevant references. A table has been added to describe all mathematical symbols along with their brief definitions. The article's grammar has been revised, and a brief description of the experimental metrics has been included. The algorithms and models from the related literature you recommended have been studied and compared. Thank you very much for your guidance!
